# Supplementary material for: Age-Related Increases in PDE11A4 Protein Expression Trigger Liquid–Liquid Phase Separation (LLPS) of the Enzyme That Can Be Reversed by PDE11A4 Small Molecule Inhibitors
Source: Cells. 2025 Jun 13;14(12):897. doi: 10.3390/cells14120897 (PMC12190369; doi:10.3390/cells14120897)
Supplement: Supplementary file 1 [file cells-14-00897-s001.zip › cells-3616488-supplementary/cells-3616488-Supplementary/Supplement Source Data Cells.pdf]

in text

Replicate	ID	[Plasmid]	PDE11A4 r.o.d.-bg/Ponceau stain r.o.d.
1	10% WT 1	10% WT	0.008963264
1	30% WT 1	30% WT	0.344083921
1	100% WT 1	100% WT	2.315097628
2	10% WT 2	10% WT	0.011297413
2	30% WT 2	30% WT	0.550304772
2	100% WT 2	100% WT	2.535093967
3	10% WT 3	10% WT	0.028738104
3	30% WT 3	30% WT	0.745129535
3	100% WT 3	100% WT	4.522076019
4	10% WT 4	10% WT	0.169291667
4	30% WT 4	30% WT	1.254870465
4	100% WT 4	100% WT	5.906249792

100%=.375ug DNA

Table1

replicate	tadalafil	cAMP CPMs-BG/ug	cGMP CPMs-BG/ug
1	GFP	61.53196741	49.83897672
1	WT	4059.917026	3921.652594
1	0.1 uM	4321.275833	3907.174712
1	1 uM	3382.726126	3505.000842
1	10 uM	2149.86138	2692.876532
1	100 uM	508.6765063	642.9869755
2	0.1 uM	4639.944338	4371.843704
2	1 uM	4146.664487	3980.111259
2	10 uM	2579.847942	2493.391608
2	100 uM	608.4237738	558.5529727
2	GFP	96.42459717	14.04102604
2	WT	3704.730107	3696.595451
3	0.1 uM	2574.831009	2309.781539
3	1 uM	4136.338765	2840.638756
3	10 uM	2627.618056	2530.583532
3	100 uM	494.9510614	546.372373
3	GFP	143.0909563	89.52668527
3	WT	3897.294829	4247.685284
4	0.1 uM	3546.902682	3478.095552
4	1 uM	4024.26825	3648.730977
4	10 uM	2797.988763	1630.355797
4	100 uM	686.0729733	398.475763
4	GFP	168.9312263	55.33618023
4	WT	3628.114029	3418.663057

replicate	ID	treatment	PDE11A4 r.o.d.-bg/Ponceau stain r.o.d.
1	DMSO1	WT DMSO	1
2	DMSO2	WT DMSO	1
3	DMSO3	WT DMSO	1
4	DMSO4	WT DMSO	1
1	0.01uM Tad 1	0.1uM Tad	1.1873
2	0.1uM Tad 2	0.1uM Tad	1.393673
3	0.1uM Tad 3	0.1uM Tad	2.018417
4	0.1uM Tad 4	0.1uM Tad	0.692544
1	1uM Tad 1	1uM Tad	1.081591
2	1uM Tad 2	1uM Tad	1.114098
3	1uM Tad 3	1uM Tad	1.994237
4	1uM Tad 4	1uM Tad	0.63065
1	10uM Tad 1	10uM Tad	1.204873
2	10uM Tad 2	10uM Tad	1.255865
3	10uM Tad 3	10uM Tad	1.390806
4	10uM Tad 4	10uM Tad	0.639624
1	100uM Tad 1	100uM Tad	0.940254
2	100uM Tad 2	100uM Tad	1.000876
3	100uM Tad 3	100uM Tad	0.940208
4	100uM Tad 4	100uM Tad	0.861286

Table1

Replicate	Sample	Treatment	cAMP CPM-bg/ug	cGMP CPM-bg/ug
1	GFP 1	GFP	133	33.5
1	DMSO 1	DMSO	2758.666667	2724.5
1	0.1uM BC11-38 1	0.1uM BC11-38	3178.333333	3372.5
1	1uM BC11-38 1	1uM BC11-38	2657	2713.833333
1	10uM BC11-38 1	10uM BC11-38	2946.333333	3082.166667
1	100uM BC11-38 1	100uM BC11-38	2273	2127.5
2	GFP 2	GFP	135.6666667	63.16666667
2	DMSO 2	DMSO	2923.666667	2892.5
2	0.1uM BC11-38 2	0.1uM BC11-38	2971.333333	3146.166667
2	1uM BC11-38 2	1uM BC11-38	3024.666667	3245.5
2	10uM BC11-38 2	10uM BC11-38	3213.666667	3059.5
2	100uM BC11-38 2	100uM BC11-38	1765.666667	1998.5
3	GFP 3	GFP	186.6666667	84.16666667
3	DMSO 3	DMSO	3021	2755.166667
3	0.1uM BC11-38 3	0.1uM BC11-38	2802	2198.166667
3	1uM BC11-38 3	1uM BC11-38	2963	2780.166667
3	10uM BC11-38 3	10uM BC11-38	3234	3093.833333
3	100uM BC11-38 3	100uM BC11-38	2172	2049.833333
4	GFP 4	GFP	164.6666667	38.5
4	DMSO 4	DMSO	2180.333333	2291.166667
4	0.1uM BC11-38 4	0.1uM BC11-38	2892.333333	2705.166667
4	1uM BC11-38 4	1uM BC11-38	2990.333333	3087.166667
4	10uM BC11-38 4	10uM BC11-38	3124.333333	2835.166667
4	100uM BC11-38 4	100uM BC11-38	1319.333333	1401.166667

Replicate	ID	Treatment	PDE11A4 r.o.d.-bg/Ponceau stain r.o.d.
1	DMSO 1	DMSO	1.08352792
1	0.1uM BC11-38 1	0.1uM BC11-38	1.45122277
1	1uM BC11-38 1	1uM BC11-38	0.794788207
1	10uM BC11-38 1	10uM BC11-38	1.476392031
1	100uM BC11-38 1	100uM BC11-38	2.086087985
2	DMSO 2	DMSO	0.91647208
2	0.1uM BC11-38 2	0.1uM BC11-38	1.117416625
2	1uM BC11-38 2	1uM BC11-38	1.378570646
2	10uM BC11-38 2	10uM BC11-38	0.597245812
2	100uM BC11-38 2	100uM BC11-38	1.109983579
3	DMSO 3	DMSO	1.103107345
3	0.1uM BC11-38 3	0.1uM BC11-38	5.172253358
3	1uM BC11-38 3	1uM BC11-38	2.163983586
3	10uM BC11-38 3	10uM BC11-38	2.251792897
3	100uM BC11-38 3	100uM BC11-38	3.632797858
4	DMSO 4	DMSO	0.896892655
4	0.1uM BC11-38 4	0.1uM BC11-38	1.811217213
4	1uM BC11-38 4	1uM BC11-38	1.296996781
4	10uM BC11-38 4	10uM BC11-38	2.113064653
4	100uM BC11-38 4	100uM BC11-38	2.967817903

data in text, representative image in Fig2A

experiment	cell # within expt	PCC	M1	M2
1	1	-0.01	0	0
1	2	0.01	2.00E-03	0.068
1	3	0	0	0.013
1	4		0	
2	1	-0.01	1.00E-03	0.013
2	2	0	2.00E-03	0.077
2	3	-0.02	2.00E-03	6.00E-03
3	1	0.01	0.015	0.079
3	2	0.03	3.00E-03	0.074
3	3	-0.03	1.00E-03	5.00E-03
3	4	-0.01	7.00E-03	0.053
4	1	-0.02	0	3.00E-03
4	2	-0.04	0.393	0.242
4	3	-0.05	0.262	0.187
4	4	0.05	0.426	0.266
4	5	0.05	0.204	0.114

data in text

HT22 experiment #	# droplets that emerge during recording	# droplets at end of recording	total minutes recorded	HT22 fusion events/hour
1*	13	5	94	5
2	4	3	42	1.42
2	23	11	50	14.4
3	8	3	60	5

total=4cells/3expts

*recording shown in Figure 2B

COS1 experiment #	# droplets that emerge during recording	# droplets at end of recording	total minutes recorded	COS1 fusion events/hour
1**	12	6	120	3
2	3	2	45	1.33
2	2	1	45	1.33
3	4	3	45	1.33
3	8	5	45	4
4	16	8	120	2
5	8	4	120	4

total = 7 cells/5 expts

**recording shown in Figure 2C

Figure 2E

replicates	Image	treatment	%Labelled Cells with Puncta	Total # counted
1	A1	vehicle	61.98347107	121
1	A2b	5% 1,6-hex	11.39240506	79
2	B1	vehicle	60	80
2	B2	5% 1,6-hex	12.28070175	57
3	C1	vehicle	67.34693878	49
3	C2	5% 1,6-hex	17.39130435	46
4	D1b	vehicle	57.89473684	114
4	D2b	5% 1,6-hex	25.97402597	77

Figure 2F

Replicate	Image	[Plasmid]	%Labelled Cells with Puncta	Total # counted
1	A2	10% WT	19.512195	82
2	B2	10% WT	14.678899	109
3	C2	10% WT	13.084112	107
4	D2	10% WT	17.525773	97
1	A3	30% WT	18.181818	132
2	B3	30% WT	26.923077	156
3	C3	30% WT	29.166667	144
4	D3	30% WT	23.376623	154
1	A5	100% WT	34.042553	188
2	B5	100% WT	30.331754	211
3	C5	100% WT	36.792453	212
4	D5	100% WT	28.776978	139

100%=.375ug DNA

Fig2G

replicate	Image	verine]/ta(	%Labelled Cells with Puncta	Total # counted
3	A3	WT	41.37931	58
2	A2	WT	42.372881	59
6	A6	WT	47.457627	59
4	A4	WT	58.333333	60
5	A5	WT	42.682927	82
4	D4	xNdis	0	46
5	D5	xNdis	5.660377	53
3	D3	xNdis	7.407407	54
2	D2	xNdis	0	57
6	D6	xNdis	3.030303	66

Figure 3D

Image	1hr tadalafil uM	%Labelled Cells with Puncta	Total # counted
A1	0	43.66197183	213
A5	0.1	50.93167702	161
A4	1	62.5	184
A3	10	16.91542289	201
A2	100	20.08733624	229
B1	0	47.89473684	190
B2	100	24.87562189	201
B3	10	20.96069869	229
B4	1	51.82926829	164
B5	0.1	40.19138756	209
C1	0	53.75722543	173
C2	100	25.98039216	204
C3	10	25.23364486	214
C4	1	43.66812227	229
C5	0.1	54.44444444	180
D1	0	47.52851711	263
D2	100	20	175
D3	10	20.90909091	220
D4	1	50	180
D5	0.1	49.45054945	182

Figure 3E

Image	tadalafil uM 1hr+5hr wash	%Labelled Cells with Puncta	tal # counted
A1	0um	54	200
A5	0.1um	54.54545455	209
A4	1um	62.43386243	189
A3	10um	55.95854922	193
A2	100um	21.42857143	210
B1	0um	38.647343	207
B2	100um	19.02985075	268
B3	10um	52.15686275	255
B4	1um	64.62264151	212
B5	0.1um	52.55813953	215
C1	0um	56.91489362	188
C2	100um	11.33603239	247
C3	10um	46.42857143	224
C4	1um	50.81967213	244
C5	0.1um	52.75229358	218
D1	0um	56.14754098	244
D2	100um	30.69767442	215
D3	10um	58.15217391	184
D4	1um	67.04545455	176
D5	0.1um	57.07317073	205

Figure 3F

Image	tadalafil 24hr	%Labelled Cells with Puncta	Total # counted
A1	0um	56.7251462	171
A5	0.1um	57.48792271	207
A4	1um	55.23809524	210
A3	10um	17.07317073	287
A2	100um	18.65284974	193
B1	0um	48	225
B2	100um	5.084745763	236
B3	10um	15.06849315	292
B4	1um	52.40384615	208
B5	0.1um	39.8340249	241
C1	0um	49.80079681	251
C2	100um	12.4	250
C3	10um	34.1991342	231
C4	1um	41.98113208	212
C5	0.1um	52.7638191	199
D1	0um	50.20746888	241
D2	100um	16.38225256	293
D3	10um	21.25	320
D4	1um	42.8057554	278
D5	0.1um	57.73809524	168

Figure 3G

Image	tadalafil uM 24hr+5hr Wash	%Labelled Cells with Puncta	Total # counted
A1	0um	54.28571429	245
A5	0.1um	59.90566038	212
A4	1um	60.64814815	216
A3	10um	41.92307692	260
A2	100um	12.04819277	332
B1	0um	52.36220472	254
B2	100um	8.960573477	279
B3	10um	38.23529412	306
B4	1um	50.20080321	249
B5	0.1um	46.21513944	251
C1	0um	48.44720497	322
C2	100um	8.620689655	232
C3	10um	39.33933934	333
C4	1um	47.25274725	273
C5	0.1um	52.47933884	242
D1	0um	45.62737643	263
D2	100um	11.5942029	345
D3	10um	38.79310345	232
D4	1um	63.24324324	185
D5	0.1um	66.97247706	218

Figure 3H

Image	BC1138 uM 1hr	%Labelled Cells with Puncta	Total # counted
A1	0um	42.23602484	161
A5	0.1um	25.65789474	152
A4	1um	36.30952381	168
A3	10um	30.1369863	146
A2	100um	29.23076923	130
B1	0um	38.99371069	159
B2	100um	18.04511278	133
B3	10um	24	150
B4	1um	38.01652893	121
B5	0.1um	50.70422535	142
C1	0um	45.18518519	135
C2	100um	13.4751773	141
C3	10um	30.81761006	159
C4	1um	30.76923077	130
C5	0.1um	40.60150376	133
D1	0um	45.73170732	164
D2	100um	25.75757576	198
D3	10um	36.73469388	196
D4	1um	50.78125	128
D5	0.1um	53.73134328	134

Figure 3I

Image	BC1138 uM 1hr+5hr Wash	%Labelled Cells with Puncta	Total # counted
A1	0	51.39664804	179
A5	0.1	50	158
A4	1	43.31550802	187
A3	10	46.47058824	170
A2	100	43.70860927	151
B1	0	52.15053763	186
B2	100	43.81443299	194
B3	10	53.01724138	232
B4	1	56.73076923	208
B5	0.1	46.66666667	195
C1	0	50	208
C2	100	46.11872146	219
C3	10	47.87234043	188
C4	1	49.68944099	161
C5	0.1	47.20812183	197
D1	0	52.65957447	188
D2	100	45.55555556	180
D3	10	47.42857143	175
D4	1	59.39086294	197
D5	0.1	54.48717949	156

Figure 3J

Image	BC1138 24h uM	%Labelled Cells with Puncta	Total # counted
A1	0um	49.32126697	221
A5	0.1um	50.89285714	224
A4	1um	38.8185654	237
A3	10um	44.97991968	249
A2	100um	22.78481013	158
B1	0um	54.49101796	167
B2	100um	26.9035533	197
B3	10um	44.7257384	237
B4	1um	47.64397906	191
B5	0.1um	33.33333333	276
C1	0um	43.05555556	216
C2	100um	18.18181818	220
C3	10um	37.8238342	193
C4	1um	48.16513761	218
C5	0.1um	40.25423729	236
D1	0um	38.42364532	203
D2	100um	24.05660377	212
D3	10um	41.75257732	194
D4	1um	48.24120603	199
D5	0.1um	49.10394265	279

Figure 3K

Image	BC1138 uM 24+5hr Wash	%Labelled Cells with Puncta	Total # counted
A1	0	45.10638298	235
A5	0.01	49.53271028	214
A4	1	48.01980198	202
A3	10	42.62295082	244
A2	100	37.5	192
B1	0	55.71428571	210
B2	100	51.30890052	191
B3	10	43.13099042	313
B4	1	39.50617284	324
B5	0.01	37.39130435	345
C1	0	49.40711462	253
C2	100	32.12435233	193
C3	10	37.97468354	316
C4	1	49.59677419	248
C5	0.01	46.59498208	279
D1	0	65.64417178	326
D2	100	34.9137931	232
D3	10	41.94630872	298
D4	1	53.44827586	174
D5	0.01	55.91397849	279

Figure 3L

replicate	Image	[rolipram]/tadalafi	%Labelled Cells with Puncta	Total # counted
5	A5	dmso	38.46153846	143
3	A3	0.1uM	34.35897436	195
2	A2	1uM	43.11377246	167
1	A1	10uM	37.33333333	150
6	A6	100uM	34.375	160
4	A4	10uMTad	8.552631579	152
1	B1	10uM	39.62264151	159
2	B2	1uM	39.67391304	184
3	B3	0.1uM	41.24293785	177
4	B4	10uMTad	7.558139535	172
5	B5	dmso	39.64497041	169
6	B6	100uM	43.39622642	159
1	C1	10uM	40.86021505	186
2	C2	1uM	38.96103896	154
3	C3	0.1uM	33.11258278	151
4	C4	10uMTad	13.19444444	144
5	C5	dmso	30.06535948	153
6	C6	100uM	33.55263158	152
1	D1	10uM	39.30635838	173
2	D2	1uM	35.48387097	155
3	D3	0.1uM	39.86013986	143
4	D4	10uMTad	15.89403974	151
5	D5	dmso	45.66473988	173
6	D6	100uM	42.06896552	145

Figure 3M

replicate	Image	[papaverine]/tadalafi	%Labelled Cells with Puncta	Total # counted
5	A5	dmso	44.73684211	152
3	A3	0.1uM	44.78527607	163
2	A2	1uM	37.41935484	155
1	A1	10uM	39.50617284	162
6	A6	100uM	42.58064516	155
4	A4	10uMTad	5.303030303	132
1	B1	10uM	41.56626506	166
2	B2	1uM	34.39490446	157
3	B3	0.1uM	28.57142857	140
4	B4	10uMTad	8.196721311	122
5	B5	dmso	43.35260116	173
6	B6	100uM	42.85714286	140
1	C1	10uM	37.6344086	186
2	C2	1uM	43.62416107	149
3	C3	0.1uM	32.51533742	163
4	C4	10uMTad	6.586826347	167
5	C5	dmso	44.23076923	156
6	C6	100uM	41.25874126	143
1	D1	10uM	39.04109589	146
2	D2	1uM	39.59731544	149
3	D3	0.1uM	36.11111111	180
4	D4	10uMTad	6.976744186	172
5	D5	dmso	42.26190476	168
6	D6	100uM	40.88050314	159

Figure 3O

ID	Treatment	PDE11A4-bg cyto/membrane r.o.d.
3	DMSO	5.331858407
3	Tadalafil	4.914691943
3	BC11-38	6.794326241
4	DMSO	2.814917127
4	Tadalafil	7.8
4	BC11-38	6.68852459
1	BC11-38	8.761006289
1	Tadalafil	6.772727273
2	BC11-38	5.208178439
2	Tadalafil	5.545112782
2	DMSO	2.692607004

Figure 4A

real ID	sex	age	group	group	# ghost axons
13413	M	O	Veh	OVeh	274
13414	M	O	11mg/kg	OD1	316
13430	M	O	110mg/kg	OD2	205
13512	F	O	Veh	OVeh	590
13519	F	O	11mg/kg	OD1	473
13520	F	O	110mg/kg	OD2	282
13521	F	O	110mg/kg	OD2	310
13522	F	O	Veh	OVeh	421
13531	M	O	Veh	OVeh	423
13533	M	O	11mg/kg	OD1	493
13536	M	O	110mg/kg	OD2	218
13361	F	O	11mg/kg	OD1	604
