# Supplementary figures and images for: Age-Related Increases in PDE11A4 Protein Expression Trigger Liquid–Liquid Phase Separation (LLPS) of the Enzyme That Can Be Reversed by PDE11A4 Small Molecule Inhibitors
Source: Cells. 2025 Jun 13;14(12):897. doi: 10.3390/cells14120897 (PMC12190369; doi:10.3390/cells14120897)

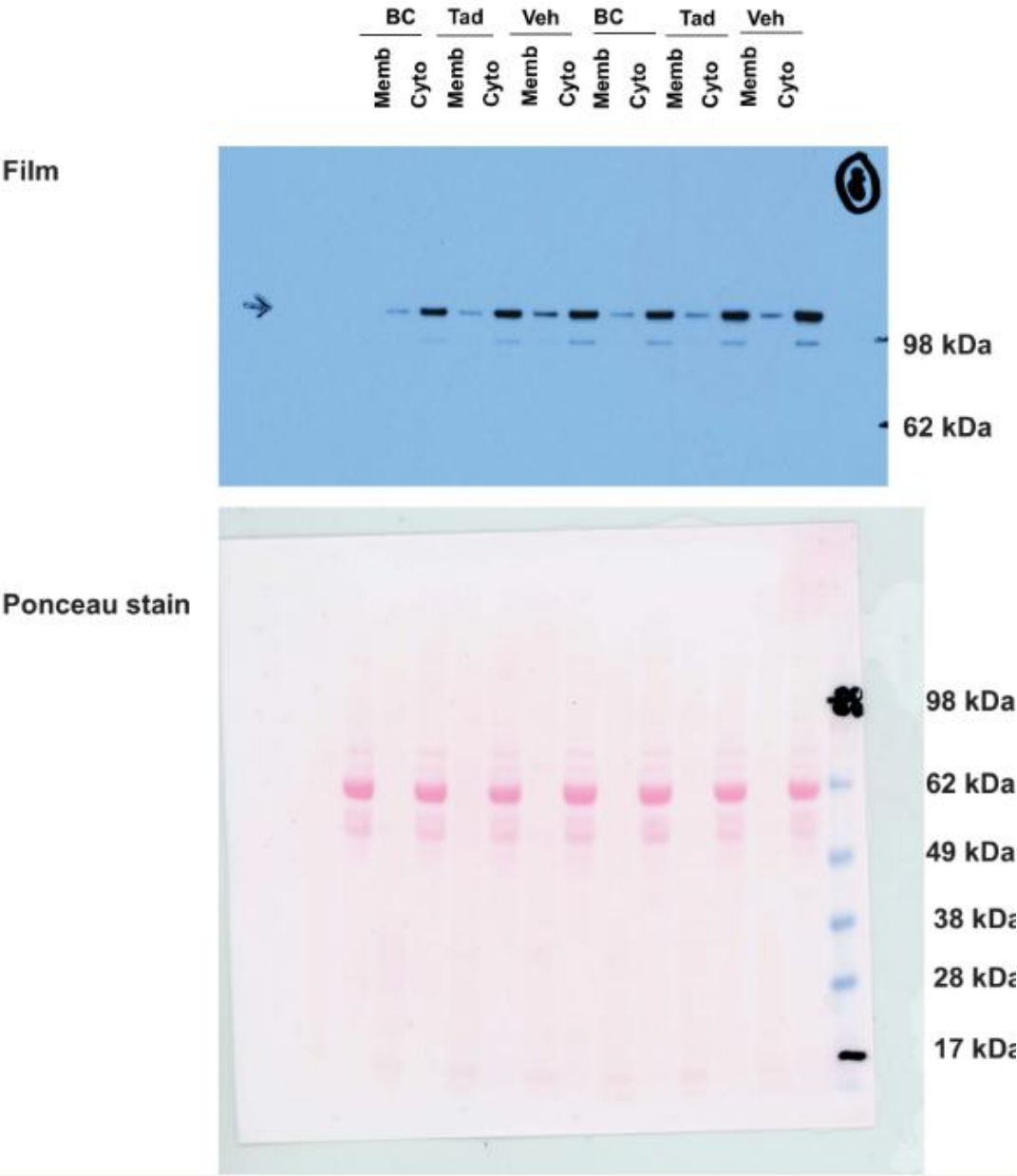

Supplement: Supplementary file 1 [file cells-14-00897-s001.zip › cells-3616488-supplementary/cells-3616488-Supplementary/Original western blot.pdf]
